# Inclusion and diversity at the *BJGP* and *BJGP Open*

**DOI:** 10.3399/BJGPO.2023.0153

**Published:** 2023-09-01

**Authors:** Euan Lawson, Hajira Dambha-Miller, Catharine Hull

**Affiliations:** 1 Editor, BJGP, London, UK; 2 Editor, BJGP Open, London, UK; 3 Head of Publishing, RCGP, London, UK

**Keywords:** Inclusion, diversity

## Introduction

The *BJGP* and *BJGP Open* are committed to the highest standards of publishing. It is natural that we pay close attention to what we are publishing. Importantly, we need to be mindful of what we are not publishing and, crucially, who we are not publishing. There are many barriers to publication but it is not right that those barriers currently include where a person is born, their gender, and their ethnicity. This is a problem that extends across academia and not just academic publishing, but we all must play our role in a cultural shift.

## The call to action

In 2017 the *Lancet* called for action to investigate gender bias in journals. An initial snapshot provided preliminary evidence of the problem.^1^ Little has changed since then. Women scientists are under-represented in the top three medical research journals and women have fewer first author publications. Over the period from 2002–2019 there was no discernible improvement in first authorship rates, with lower citation counts and fewer co-authors.^2^ There may be slightly better representation of women in academic primary care compared to general medicine.^3^ Similarly, the problems of structural racism are felt across academic medicine, and medical journals are no exception.^^4^^

The Royal College of General Practitioners (RCGP) is a member of the Joint Commitment for Action on Inclusion and Diversity in Publishing.^5^ This group, launched in June 2020, has brought together scholarly publishers from across the globe and a wide range of disciplines. There are now 56 organisations with responsibility for over 15 000 journals participating. The group acknowledges *‘... that biases exist in scholarly publishing and we commit to scrutinising our own processes to minimise these. We will pool our resources, expertise and insight to accelerate research culture change’*.

## Minimum standards

The Joint Commitment group has set six minimum standards for inclusion and diversity in scholarly publishing^6^ and these are presented in Box 1. We are fully supportive of these standards. We also recognise they are the minimum standards and represent the start of the process.^6^ There is much work to do and there is a need to ensure we use this lens when we consider the varied activities of the journals.

Box 1 **Six minimum standards for inclusion and diversity in scholarly publishing^6^**

1. Ensure inclusion and diversity are integrated into publishing activities and strategic planning.

2. Work to understand the demographic diversity of authors, editorial decision makers, and reviewers, such as gender, geography, and ethnicity data.

3. Acknowledge the barriers within publishing that authors, editorial decision makers, and reviewers from under-represented communities experience and take actions to address them.

4. Define and communicate the specific responsibilities authors, editorial decision makers, reviewers, and staff members have towards inclusion and diversity.

5. Review and revise as appropriate the appointment process for editors and editorial boards to capture the widest talent pool possible.

6. Publicly report on progress on inclusion and diversity in scholarly publishing at least once a year.

The Joint Commitment group has agreed a set of questions on race, ethnicity, and gender,^7^ and worked with peer review systems to be able to collect these anonymised data and hold them in a way that meets privacy and GDPR legislation. These data are self-reported, meaning that a corresponding author cannot answer these questions on behalf of a co-author. The data categorisation has come from the Joint Commitment group and undergone extensive testing and validation, including input from patients and the public.

## Changes to the *BJGP* and *BJGP Open*

Like others in the Joint Commitment group, we will now be rolling this out across *BJGP* and *BJGP Open* from 1 September 2023. This will be the start of our data collection and a major step in our commitment towards being an inclusive journal. All our authors and reviewers will now have the option to complete information on race, ethnicity, and gender when accessing the ScholarOne system for *BJGP* and *BJGP Open*. These categorisations have been agreed by the Joint Commitment group and undergone extensive testing and validation, and have already been rolled out by a number of large publishers with multiple journals across many disciplines, including medicine and health care. These standardised lists will be incorporated into our systems. It should be noted that answering these questions is voluntary, and while the questions cannot be skipped, each question has a ‘Prefer not to disclose’ option.

No individual level data will ever be accessible to anyone at *BJGP* journals at any time. Accordingly, the normal editorial processes of *BJGP* and *BJGP Open* will continue and there is no mechanism where these data can play any part in editorial decisions. The data will be held securely on the ScholarOne system, and the process and privacy policies have been agreed by the RCGP’s Data Protection Officer and Legal Counsel.

These aggregated and anonymised data will be used to ensure we are meeting the Joint Commitment group’s six minimum standards for inclusion and diversity in scholarly publishing.^6^ By all publishers in the Joint Commitment group using the same standardised questions, over time, we are aiming to have a data-driven approach to inform our goals around diversity, inclusion, and equity in scholarly communications and research more broadly. We commit to collating these data for annual review at the respective journal Editorial Board meetings and we welcome their input. Furthermore, we commit to openly sharing these aggregated anonymised data with our readers and ensuring they are available to the public.

We will be prepared to respond to whatever we find from our data collection in a way that is open, honest, proportionate, and meaningful. We will develop our response together with our readers and with oversight from the Editorial Boards to ensure that no one is disadvantaged by the findings.

We will update you with our progress and invite your comments and feedback as we navigate this new endeavour as a journal.

**Euan Lawson,**

Editor, *BJGP*.

**Hajira Dambha-Miller,**

Editor, *BJGP Open*.

**Catharine Hull,**

Head of Publishing, RCGP, London.
